# Bilateral Testicular Infarction from IgA Vasculitis of the Spermatic Cords

**DOI:** 10.1155/2017/9437965

**Published:** 2017-11-21

**Authors:** Mazen Toushan, Ashka Atodaria, Stephen D. Lynch, Hassan D. Kanaan, Limin Yu, Mitual B. Amin, Mamon Tahhan, Ping L. Zhang, Paul S. Kellerman, Abhishek Swami

**Affiliations:** ^1^Division of Anatomic Pathology, Department of Pathology, Beaumont Health, Royal Oak, MI, USA; ^2^Department of Internal Medicine, Beaumont Health, Royal Oak, MI, USA; ^3^Division of Nephrology, Department of Internal Medicine, Beaumont Health, Royal Oak, MI, USA

## Abstract

A 51-year-old man with type 2 diabetes mellitus and chronic obstructive pulmonary disease presented to the emergency room with increasing bilateral leg pain, rash, and scrotal swelling with pain. Skin biopsy from his thigh revealed IgA-associated vasculitis. Due to hematuria, a renal biopsy was performed and showed an IgA glomerulonephritis with focal fibrinoid necrosis and neutrophil accumulation. Bilateral orchiectomies were performed in two separate procedures ten and thirteen days after the renal biopsy, as a result of uncontrolled abscess formation in testicles. Microscopically, both testicles revealed large abscess formation destroying almost the entire testicular parenchyma without tumor cells. Spermatic cord margins were further scrutinized microscopically to show bilateral vasculitis in many small size vessels, confirmed by positive endothelial staining for IgA. Some of the affected arteries revealed central organizing thrombi with recanalization features, highly suggestive of vasculitis-associated thrombi formation, resulting in testicular ischemic infarction and abscess formation. We conclude that this adult patient developed a severe form of Henoch-Schönlein purpura, with vasculitis affecting multiple organs, including the most serious and unusual complication of bilateral testicular infarction.

## 1. Introduction

Henoch-Schönlein purpura (HSP) is a systematic vasculitis presenting primarily in children, but less so in adults, often resulting in IgA-associated vasculitis in skin and IgA nephritis [1, 2]. HSP can also present with arthritis, gastrointestinal bleeding, and orchitis with symptoms of testicular pain and swelling in up to 20% of affected boys clinically [3–9], but there has been no pathologically proven IgA-associated vasculitis of the testicles documented even in patients with testicular pain. In addition, IgA-associated orchitis has not been previously described in adults. We report an unusual case in a 51-year-old man who developed IgA-associated vasculitis involving the skin, kidneys, and bilateral spermatic cords resulting in bilateral testicular infarction. This is the first report of histologically proven IgA-associated orchitis in the literature.

## 2. Case Presentation

A 51-year-old male with uncontrolled diabetes (type II) presented to the hospital with severe lower extremity and scrotal edema, associated with pain, and extremity rash. The rash began 3 weeks prior to presentation and involved his lower abdomen, bilateral lower extremities, and scrotum. He reported intermittent painful edema of his legs and scrotum for the past year which had been attributed to neuropathic pain related to uncontrolled diabetes and chronic venous stasis. Patient also reported fatigue, malaise, 50-pound weight loss over the past one year, intermittent bloody bowel movements, and dysuria but denied any fevers, chills, hematuria, history of sexually transmitted infections, HIV, or malignancy.

Two weeks prior to presentation at our hospital, the patient had presented to an outside hospital with syncope and was found to be hypoglycemic. Biopsy of the rash from his calf was positive for leukocytoclastic vasculitis. Autoimmune workup was negative except for elevated C-reactive protein (CRP). Bilateral lower extremity Doppler study was negative for thromboembolism. Patient was treated with Vancomycin followed by clindamycin for cellulitis. He left against medical advice before presenting to our hospital.

The patient's past medical history was positive for uncontrolled diabetes type II with peripheral neuropathy, peripheral vascular disease with chronic lower extremity ulcers, chronic obstructive pulmonary disease, and opioid dependence. His past surgeries included amputation of a digit on his right hand due to osteomyelitis with gangrene and lumbar spinal fusion. Family history was positive for breast cancer in his sister. There was no family history of autoimmune disease. He is a current smoker with a 40-pack-year history and denied any current alcohol or drug use. His medications included basal-bolus insulin, glipizide 10 mg twice daily, furosemide 40 mg twice daily, pregabalin, and methadone maintenance.

At presentation, the patient was found to be afebrile with blood pressure of 151/105 mmHg, heart rate of 88/per minute, respiratory rate of 20/per minute, SpO2 of 94%, and BMI of 22.8. His laboratory indices are presented in Table 1. The patient appeared to be cachectic with peripheral wasting. Exam revealed tachycardia with regular rhythm and no murmurs. Lung exam revealed wheezes bilaterally. His abdomen was distended and tender to palpation. There was tender scrotal edema as well as severe pitting edema of his lower extremities. He had a diffuse purpuric rash over his lower extremities, genitalia, and abdomen. Smaller petechiae were found on his hands and arms. He also had multiple healing lesions on his legs, a chronic healing ulcer under the left heel, and a large ulcer with eschar without drainage or odor on the right lower leg.

Records from his previous admission showed elevated CRP serology, and autoimmune work was negative. A skin biopsy of the lower extremity rash done at an outside hospital was positive for leukocytoclastic vasculitis.

His chest X-ray was negative and ECG was unremarkable. Scrotal ultrasound showed bilateral wall edema with inguinal lymphadenopathy. CT of the abdomen and pelvis also showed anasarca with bilateral inguinal and para-aortic and external iliac lymphadenopathy. CT and ultrasound of the kidneys were unremarkable. Lower extremity Doppler was negative for deep vein thrombosis.

Repeat rheumatological workup revealed positive ANA with a titer of 1 : 320 and negative anti-dsDNA, Smith, RNP, Sjogren SSA, and SSB. C3 and C4 levels were normal. Serum immunoglobulins revealed elevated IgA level at 431 mg/dL with normal IgG and IgM. Serum protein electrophoresis showed elevated kappa and lambda light chains and low albumin with elevated alpha 1 and beta globulins, suggestive of active inflammation. Blood, urine, and stool cultures were negative. The patient tested positive for* C. difficile* stool antigen. EGD and colonoscopy were performed which were negative for malignancy and hemolytic workup was negative.

Patient was initially started on IV Vancomycin for sepsis, scrotal elevation, glucose control with basal-bolus insulin, and local wound management. Vancomycin was held upon negative cultures. Patient was started on intravenous solumedrol 30 mg every 8 hours for vasculitis. Pain control with pregabalin, patient controlled analgesia (PCA) pump, and total parenteral nutrition (TPN) were initiated.

**Figure 1:**
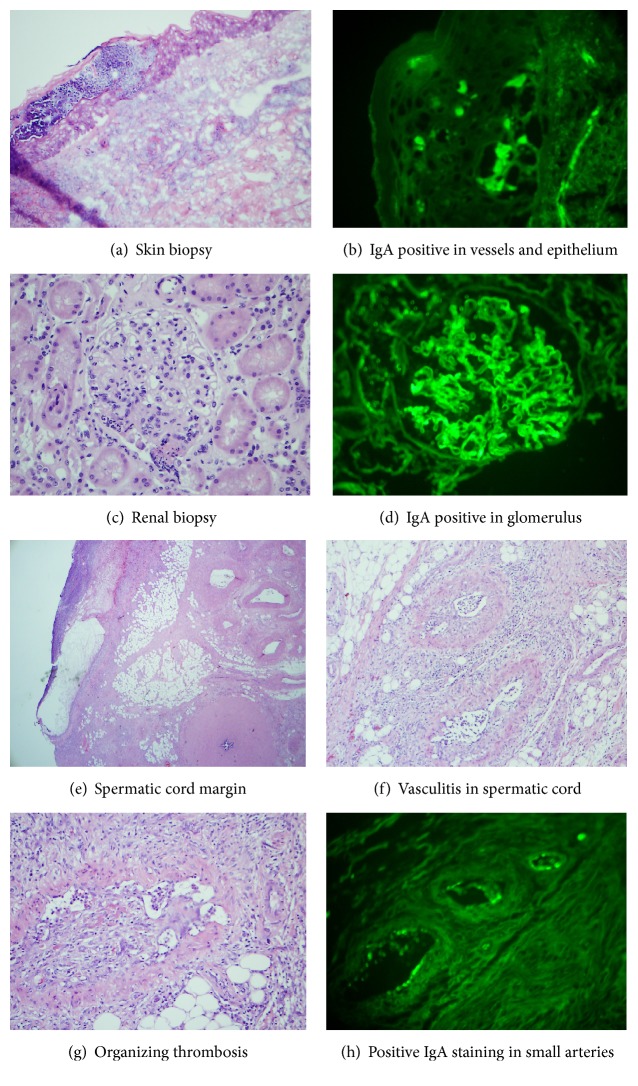
Evaluation of skin biopsy, renal biopsy, and orchiectomy specimens from the 51-year-old man. (a) Hematoxylin and eosin stained section revealed surface ulceration in the skin. (b) IgA immunofluorescence was positive in epidermis and vessels of dermis. (c) Hematoxylin and eosin stained section revealed mesangial expansion with focal neutrophil aggregation in the glomerulus. (d) IgA immunofluorescence was positive mainly in the mesangium and some along the glomerular capillary loops. (magnifications ×400 in (a)–(d)). (e) A low power view (×40) revealed the unremarkable vas deferens at the right lower corner and necrosis and abscess in the testicular parenchyma at the left upper corner. (f) Vasculitis was seen in multiple small arteries of spermatic cord at medium power view (×200). (g) High power view (×400) revealed organizing thrombus in a small artery causing nearly total occlusion of the vessel in the spermatic cord. (h) IgA immunofluorescence (×200) was positive (green granular staining) at the endothelium of multiple inflamed small arteries. Hematoxylin and eosin stains were performed in (e)–(g).

The patient's purpuric rash improved significantly and rapidly with intravenous solumedrol, but his scrotal pain and edema persisted and patient developed painful penile ulcer. A repeat skin biopsy was performed from his left thigh which showed leukocytoclastic vasculitis. Immunofluorescence was positive for IgA, IgM, and C3 in the vessel wall of the superficial dermis, consistent with IgA-associated leukocytoclastic vasculitis (Figures 1(a) and 1(b)).

His serum creatinine levels were at 0.83 to 1.1 mg/dL, but his urine analysis revealed 3+ blood and 2-3+ protein, and the protein/creatinine ratio was 1.6. A subsequent 24-hour urine protein was 2338 mg/24 hours. He was evaluated by nephrologist and a renal biopsy was performed. Light microscopy revealed two cores of renal tissue. Eleven glomeruli were identified without evidence of diffuse proliferation, crescents, and global or segmental sclerosis. Many of the glomeruli showed an increase in mesangial cellularity with focal neutrophilic infiltration as well as fibrinoid necrosis. The glomerular basement membrane showed no significant microscopic abnormality (Figure 1(c)). Masson trichrome stain revealed minimal to mild interstitial fibrosis. The blood vessels were mildly thickened without vasculitis or thrombi. Immunofluorescence study showed 3+ positive granular IgA staining in the mesangial and peripheral loop of the glomeruli (Figure 1(d)). There was mesangial and peripheral granular staining for IgM at 1+, C3 at 1+, kappa at 1+, and lambda at 2+, while IgG and C1q stained negatively in the glomeruli. Ultrastructurally, there was focal effacement of foot processes. The basement membranes were slightly thickened. Scattered immune complex deposits were identified in the mesangial areas but not in subendothelial spaces or subepithelial areas. The overall findings supported a diagnosis of IgA glomerulonephritis. Because there were no history of staphylococcal infection and no diffuse proliferative pattern in the glomeruli, with no “humps” identified at subepithelial spaces, a potential differential diagnosis of IgA dominant postinfectious glomerulonephritis was excluded [10, 11].

Repeat ultrasound of the scrotum with Doppler was done due to persistent scrotal pain, which revealed hypoperfusion of the left testicle without evidence of torsion. Left orchiectomy was performed 3 weeks after admission. Grossly the cut surface of the testicle revealed the entire testicular parenchyma to be brown-red, partially liquefied, and necrotic. In the area of the epididymis and rete testes there was yellow-green soft discoloration. The microscopy sections revealed testicular infarction with testicular/paratesticular abscess that involved the epididymis and spermatic cord.

Pain in his right testicle persisted, and, four days later, orchiectomy of the right testicle was also performed. Both gross and microscopic findings in the right testicle were similar to those in the left orchiectomy specimen. Testicular abscess was identified (Figure 1(e)). Spermatic cord margins from bilateral orchiectomy specimens were further analyzed to show diffuse vasculitis in small arteries with scattered organizing thrombi in some (Figures 1(f) and 1(g)). Paraffin embedded sections of bilateral spermatic cords were digested and stained for IgA by direct immunofluorescent method (as previously reported) [12]. The immunofluorescent section revealed strongly positive IgA staining along the endothelium of inflamed small arteries (Figure 1(h)), confirming the IgA vasculitis of the spermatic cords as the cause of the testicular ischemic infarction. His scrotal edema gradually improved with wound care and nutritional support. In addition to steroids, dapsone was started per Rheumatology.

His hospital course was complicated by persistent diarrhea, drug-seeking behavior, bacteremia, persistent hyperglycemia, and ischemia of multiple digits requiring amputations. His rash did not recur while on the steroids, and he was discharged to a long-term acute care facility with close follow-up.

## 3. Discussion

Scrotal manifestations of HSP are overwhelmingly described in pediatric populations, based solely on clinical evaluations [3–9]. No histologically proven cases of IgA-associated orchitis have been reported in any pediatric study. Furthermore scrotal disease due to IgA vasculitis is easily missed or misdiagnosed due to low level of suspicion and its propensity to manifest later in the course of disease, sometimes after initial signs and symptoms of HSP have resolved. HSP in adults is usually associated with worse outcomes compared to children [1, 2]. It is unclear whether IgA-associated orchitis in adults would have worse outcomes compared to children. In this patient the involvement was severe leading to tissue necrosis and required bilateral orchiectomies despite high dose steroid therapy. Due to initial lack of awareness the etiology for the patient's scrotum swelling had remained uncertain and was felt to be part of generalized edema and nephrotic syndrome. Patient developed* Klebsiella* bacteremia and the source of this bacteremia was felt to be from extremity ulcers and soft tissue infections. Later it was realized only after orchiectomy that the source of bacteremia was most likely from testicular infarction.

During the examination of the spermatic cords, vasculitis, characterized by edematous changes in vessel walls and infiltration of inflammatory cells, was seen in small arteries with occasional organizing thrombi. In addition, we reprocessed the paraffin embedded tissue for immunofluorescent staining of IgA, and IgA positivity was only present along endothelium of inflamed vessels, confirming that the IgA-associated vasculitis was the etiology causing thrombotic obstruction in the vessels with subsequent ischemic pain in the scrotum, testicular infarction, abscess formation, and possible overgrowth of bacteria.

In summary, this 51-year-old male patient developed a systemic IgA vasculitis involving the skin of the extremities, kidneys, and bilateral testicles with the most serious complication of testicular infarctions and subsequent abscess formation. This is the first report of histologically proven IgA-associated orchitis in the literature. This case illustrates the need for low threshold of suspicion for vasculitic scrotal involvement when caring for adult patients with HSP who develop scrotal pain and swelling. Scrotal involvement may be more prevalent than reported. Genital examination is often not performed; also scrotal pain may be mislabeled, which may lead to diagnosis being missed. Genital examination should be routinely carried out in these patients for early detection of scrotal involvement. Scrotal swelling, pain, and tenderness should prompt immediate diagnostic evaluation and urology consultation where needed.

## Figures and Tables

**Table 1:** Patient's laboratory values upon admission.

Component	Value	Ref range & units
Complete blood count with differential		
WBC	5.7	3.5–10.1 bil/L
RBC	3.56 (L)	4.31–5.48 tril/L
Hemoglobin	9.5 (L)	13.5–17.0 g/dL
Hematocrit	32.0 (L)	40.1–50.1%
MCV	90	80–100 fL
MCH	27 (L)	28–33 pg
RDW CV	17 (H)	12–15%
Platelets	395	150–400 bil/L
Neutrophils	4.4	1.6–7.2 bil/L
Lymphocytes	0.7 (L)	1.1–4.0 bil/L
Monocytes	0.4	0.0–0.9 bil/L
Immature granulocytes	0.07 (H)	0.00–0.04 bil/L

Urine analysis		
Color	Yellow	
Clarity	Clear	
Glucose	+3	Negative
Protein	+2	Negative
Blood	trace	Negative
Ketones	negative	Negative
RBCs	4–10/hpf	0–3/hpf
WBCs	5–10/hpf	0–5/hpf
Casts, hyaline	0–2/lpf	0–2/lpf
Urine protein to creatinine ratio	1.6	0–0.2

Blood chemistries		
Sodium	127 (L)	135–145 mmol/L
Potassium	6.0 (H)	3.5–5.2 mmol/L
Chloride	96 (L)	98–110 mmol/L
Carbon dioxide (CO2)	22	22–32 mmol/L
Anion gap	9	5–17
Glucose	700 (HH)	60–99 mg/dL
Blood urea nitrogen (BUN)	33 (H)	8–22 mg/dL
Creatinine	1.18	0.60–1.40 mg/dL
Calcium	7.0 (L)	8.4–10.4 mg/dL
Protein total	5.2 (L)	6.4–8.6 g/dL
Albumin	1.7 (L)	3.5–5.1 g/dL
Globulin	3.5	2.2–4.0 g/dL
Albumin/globulin ratio	0.5	
Alkaline phosphatase (ALP)	71	30–110 U/L
Aspartate aminotransferase (AST)	38 (H)	10–37 U/L
Alanine aminotransferase (ALT)	23	9–47 U/L
Bilirubin total	0.9	0.3–1.2 mg/dL
Bilirubin direct	0.3	0–0.3 mg/dL
GFR non-African American	71	>59 mL/min/1.73 m2
GFR African American	82	>59 mL/min/1.73 m2
ESR	46 (H)	0–15 mm/hr
Lactic acid	3.1	0.5–2.2 mmol/ L
Lipase	10	7–60 U/L
Beta hydroxybutyrate	0.10	0.02–0.27 mmol/L
BNP	51	0–100 pg/mL
